# Inhibition of Glucosyltransferase Activity and Glucan Production as an Antibiofilm Mechanism of Lemongrass Essential Oil against *Escherichia coli* O157:H7

**DOI:** 10.3390/antibiotics9030102

**Published:** 2020-02-29

**Authors:** Luis A. Ortega-Ramirez, M. Melissa Gutiérrez-Pacheco, Irasema Vargas-Arispuro, Gustavo A. González-Aguilar, Miguel A. Martínez-Téllez, J. Fernando Ayala-Zavala

**Affiliations:** Centro de Investigación en Alimentación y Desarrollo, A. C. Carretera Gustavo Enrique Astiazarán Rosas, No. 46. La Victoria, C. P. 83304. Hermosillo 83000, Sonora, Mexico; ingeluis_100@hotmail.com (L.A.O.-R.); melissa.gtzpacheco@gmail.com (M.M.G.-P.); iris@ciad.mx (I.V.-A.); gustavo@ciad.mx (G.A.G.-A.); norawa@ciad.mx (M.A.M.-T.)

**Keywords:** extracellular polymeric substance matrix, cellulose synthesis, enzyme inhibition, essential oils

## Abstract

The resistance of *Escherichia coli* O157:H7 to disinfection is associated with its ability to form biofilms, mainly constituted by glucans produced by glucosyltransferases. Citral and geraniol, terpenes found in the essential oil of *Cymbopogon citratus* (EO), have proven antibacterial activity against planktonic *E. coli*; however, no information was found about their efficacy and mode of action against *E. coli* biofilms. Therefore, the inhibitory effect of *C. citratus* EO, citral, and geraniol on glucans production and glucosyltransferase activity as anti-biofilm mechanism against *E. coli* was evaluated. EO, citral, and geraniol inhibited the planktonic growth of *E. coli* (minimal inhibitory concentration or MIC= 2.2, 1.0, and 3.0 mg/mL, respectively) and the bacterial adhesion (2.0, 2.0, and 4.0 mg/mL, respectively) on stainless steel. All compounds decreased the glucans production; citral and geraniol acted as uncompetitive inhibitors of glucosyltransferase activity (The half maximal inhibitory concentrations or IC_50_ were 8.5 and 6.5 µM, respectively). The evidence collected by docking analysis indicated that both terpenes could interact with the helix finger of the glucosyltransferase responsible for the polymer production. In conclusion, *C. citratus* EO, citral, and geraniol inhibited glucosyltransferase activity, glucans production, and the consequent biofilm formation of *E. coli* O157:H7.

## 1. Introduction

The persistence and the resistance of *Escherichia coli* O157:H7 to disinfection are associated with its ability to form biofilms on food contact surfaces. Biofilms are communities of microorganisms embedded in an aqueous matrix of extracellular polymeric substances (EPS) produced by the attached cells; EPS are mainly composed by polysaccharides, proteins, lipids, and nucleic acids, which can vary in composition among strains and environmental conditions [1]. The adhesion and the biofilm formation of *E. coli* on food contact surfaces causes cross-contamination, and its consequences are observed on continuous outbreaks every year [2]. It has been reported that *E. coli* O157:H7 biofilms on stainless steel can lead to the release of embedded cells to contaminate other surfaces [3]. This information highlights the importance of studying the characteristics of *E. coli* biofilms to assure effective disinfection procedures.

Exopolysaccharides are secreted during *E. coli* O157:H7 biofilm development, and some of them include cellulose, colanic acid, and the adhesin poly-β-1,6-N-acetyl-glucosamine, and these polymers are involved in the maintenance of biofilm structure and cellular protection against disinfectants [4]. It has been reported that cellulose is the major EPS component of *E. coli* biofilms, and it is essential for its structure and strength, creating cell–cell and cell–surface interactions, retaining water, and avoiding the effect of disinfectants [5]. Previously it was demonstrated that degradation of the EPS matrix of *E. coli* O157:H7 biofilms (mainly composed by glucans) increased their susceptibility to disinfectants. The synthesis and the secretion of glucans are carried out by the enzyme glucosyltransferase, consisting of three transmembrane proteins (BcsA, BcsB, and BcsC) [6]. BcsA is the catalytically active subunit located within the cell, and it is responsible for the uridine diphosphate glucose (UDP-glucose) condensation, then the product is transferred to BcsB and BcsC subunits for processing and extracellular secretion [6]. Thus, blocking this enzymatic process could lead to the inhibition of biofilm production, leaving planktonic *E. coli* more susceptible to disinfectants. 

The essential oil (EO) of lemongrass (*Cymbopogon citratus*) is rich in terpenes such as citral (85%) and geraniol (1.5%). *C. citratus* EO has been effective in inhibiting the planktonic growth of *E. coli* O157:H7 with a minimal inhibitory concentration (MIC) of 0.63 mg/mL [7], while Singh et al. [8] reported an MIC value of 0.008 mg/mL. On the other hand, citral and geraniol also showed antibacterial activity against *E. coli* as well as anti-quorum sensing activity at concentrations of 0.01 and 0.06 mg/mL, respectively [9]. On the other hand, *C. citratus* EO in combination with *Allium cepa* EO reduced the presence of *E. coli* in lettuce and spinach [10]. However, its antibacterial activity on planktonic cells could differ from the expected response against biofilms. In addition, *C. citratus* EO was able to inhibit *Staphylococcus aureus* and *Streptococcus mutans* biofilms [11,12]. Previous evidence described the ability of citral and geraniol-like terpenes to traverse the bacterial membrane and interact with vital metabolic enzymes [13]. Previous studies also evidenced the potential of citral to inactivate several enzymes [14,15]. Therefore, the objective of this study was to explore the effect of *C. citratus* EO, citral, and geraniol on the glucans production, glucosyltransferase activity, and biofilm formation of *E. coli* O157:H7.

## 2. Results

### 2.1. Susceptibility of Planktonic and Biofilm E. coli O157:H7 Cells to C. citratus EO, Citral, and Geraniol

**Figure 1:**
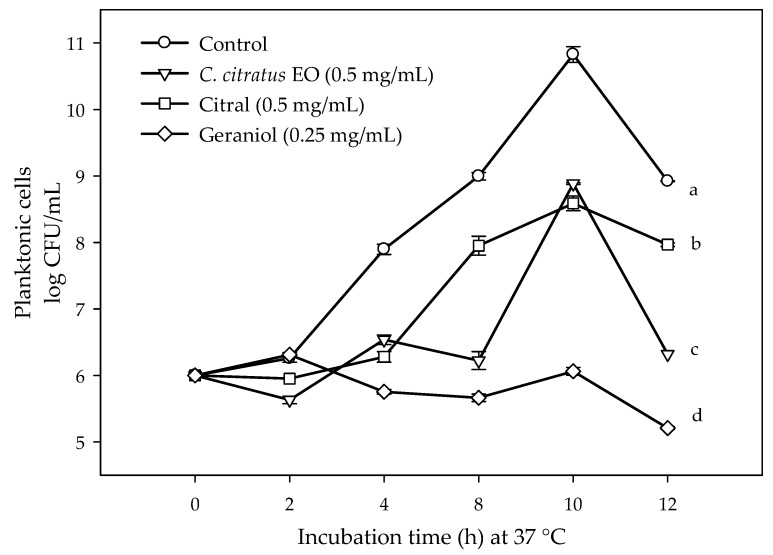
Viability changes of planktonic *E. coli* O157:H7 exposed to non-lethal concentrations of *C. citratus* essential oil (EO), citral, and geraniol. Different letters among treatments indicated significant differences among them (*p* < 0.05). The values are means ± SD, *n* = 3.

Citral was effective inhibiting the growth of planktonic cells (minimal inhibitory concentration or MIC = 1.0 mg/mL) compared to biofilm (minimal biofilm inhibitory concentration or MBIC = 2.0 mg/mL), followed by *C. citratus* EO [MIC = 2.2 mg/mL and MBIC = 2.0 mg/mL] and geraniol (MIC = 3.0 mg/mL and MBIC = 4.0 mg/mL). In general, higher concentrations were needed to inhibit the cell adhesion and the biofilm formation compared to those needed to inhibit the growth of planktonic cells. Lower concentrations than the MIC and the MBIC values of the treatments (*C. citratus* EO = 0.5 mg/mL, citral = 0.5 mg/mL, geraniol = 0.25 mg/mL) were selected to avoid interference of the loss of viability on the biofilm formation and glucans production responses (Figure 1). 

**Figure 2:**
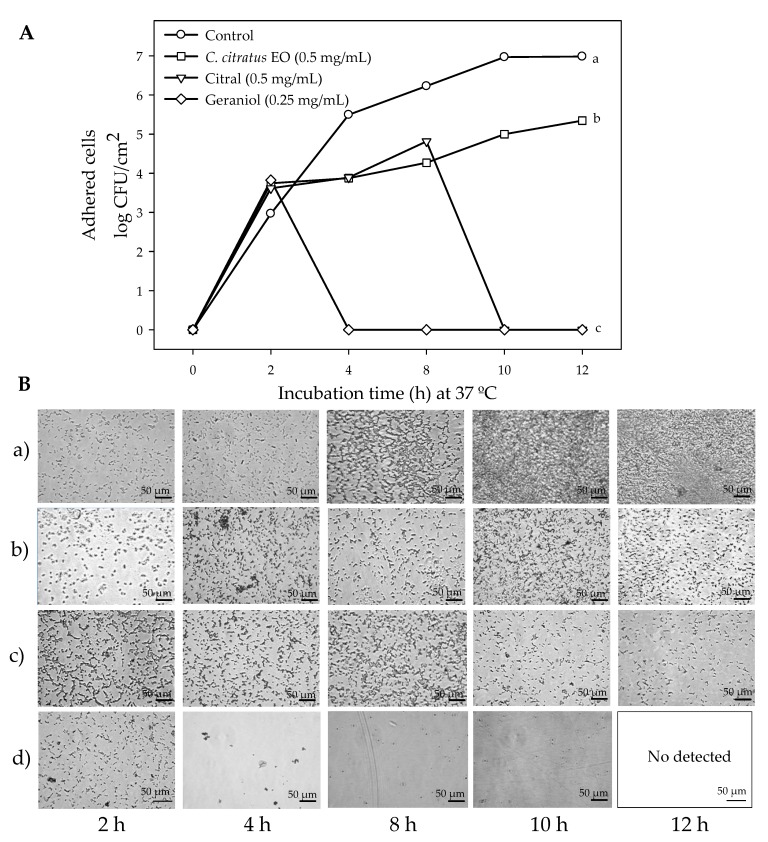
(**A**) Viability changes of biofilm embedded *E. coli* O157:H7 cells exposed to non-lethal concentrations of *C. citratus* EO, citral, and geraniol; different letters indicate significant differences among average of treatments (*p* < 0.05). The values are means ± SD, *n*= 3. (**B**) Light microscopy analysis of *E. coli* O157:H7 biofilms: (a) control, (b) *C. citratus* EO, (c) citral, (d) geraniol. Microphotographs were captured at 600x magnification in an Axio-Vert Microscope.

Figure 2A shows the *E. coli* O157:H7 biofilm cells on stainless steel coupons in the presence of the treatments. It can be observed that the viable cell in the control bacteria increased steadily as a function of the incubation time, reaching a maximum growth at 12 h at 37 °C. On the other hand, *C. citratus* EO, citral, and geraniol significantly reduced biofilm cells on stainless steel surfaces. *C. citratus* EO reduced 1.64 log CFU/cm^2^ the cell adhesion at 12 h compared to the control bacteria, whereas citral and geraniol completely inhibited cell adhesion at the end of incubation time. Figure 2B shows the microphotographs of *E. coli* biofilm development at different incubation times in the absence and presence of the compounds. A significant increase in bacterial aggregation was observed in the control (a) as the incubation time increased, being at 10 h a complete surface colonization. In the case of *C. citratus* EO (b), we observed a significant reduction in aggregation compared to the control, keeping constant at 10 and 12 h, whereas for citral (c), a significant reduction was observed after 8 h. Geraniol (d) completely inhibited *E. coli* biofilm formation, since no bacterial aggregation was observed after 2 h of incubation at 37 °C.

### 2.2. Effect of C. citratus EO, Citral, and Geraniol on the Glucans Content in E. coli O157:H7 Biofilms

**Figure 3:**
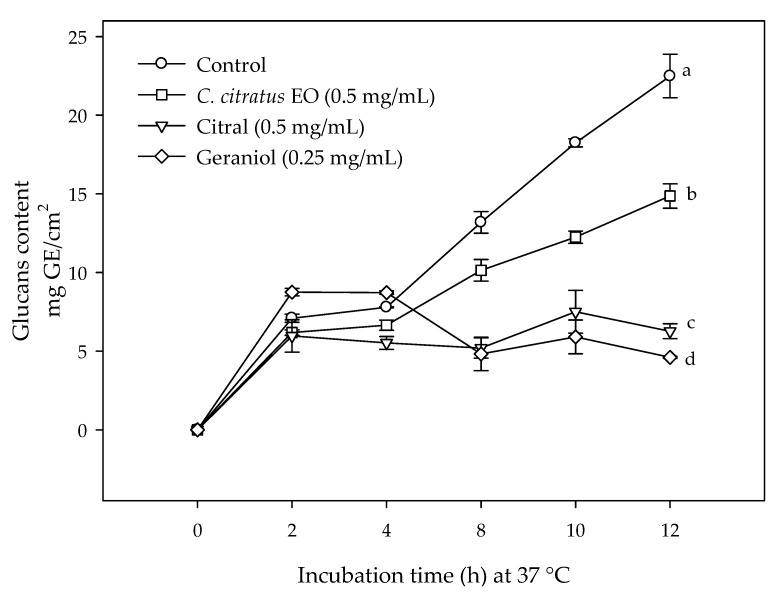
Glucans content on stainless steel coupons produced by *E. coli* O157:H7 biofilms exposed to non-lethal concentrations of *C. citratus* EO, citral, and geraniol; different letters indicate significant differences among treatments (*p* < 0.05). The values are means ± SD, *n* = 3.

Figure 3 shows the glucan content in *E. coli* biofilms exposed to *C. citratus* EO (0.5 mg/mL), citral (0.5 mg/mL), and geraniol (0.25 mg/mL). It can be observed that the glucan content of control increased exponentially during the incubation time, whereas in those treated with *C. citratus* EO, citral, and geraniol, the glucans production during the biofilm formation was significantly reduced. The stainless steel coupons exposed to citral and geraniol had a lower glucan content compared to the control and the *C. citratus* EO treated bacteria. The relationship between the secreted glucans and the biofilm cells on stainless steel surfaces showed a Pearson correlation coefficient of 0.768 with a probability of 0.0000119. 

### 2.3. Inhibition of Glucosyltransferase Activity by Citral and Geraniol

**Figure 4:**
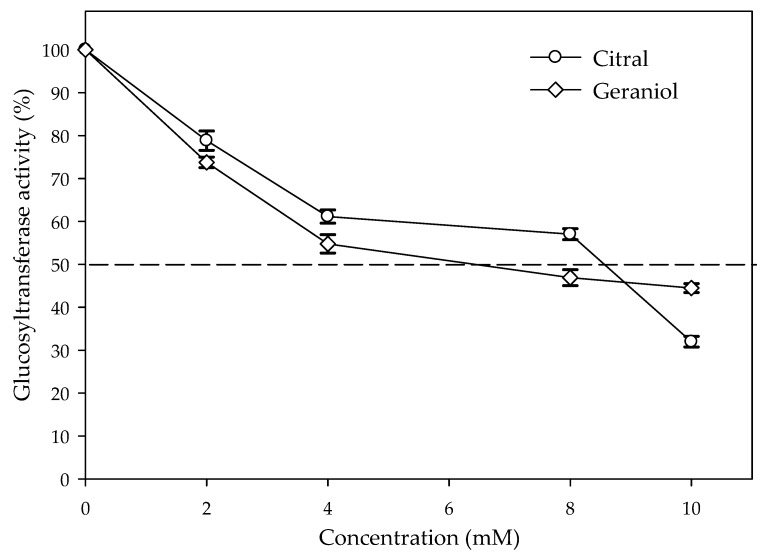
Glucosyltransferase inhibition by the presence of citral and geraniol at different. concentrations (*p* < 0.05). The values are means ± SD, *n* = 3.

**Figure 5:**
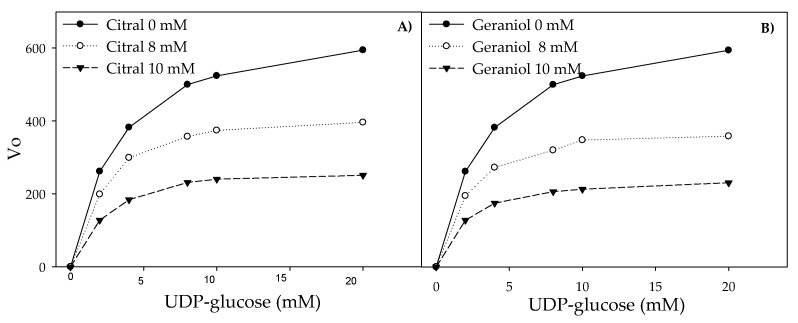

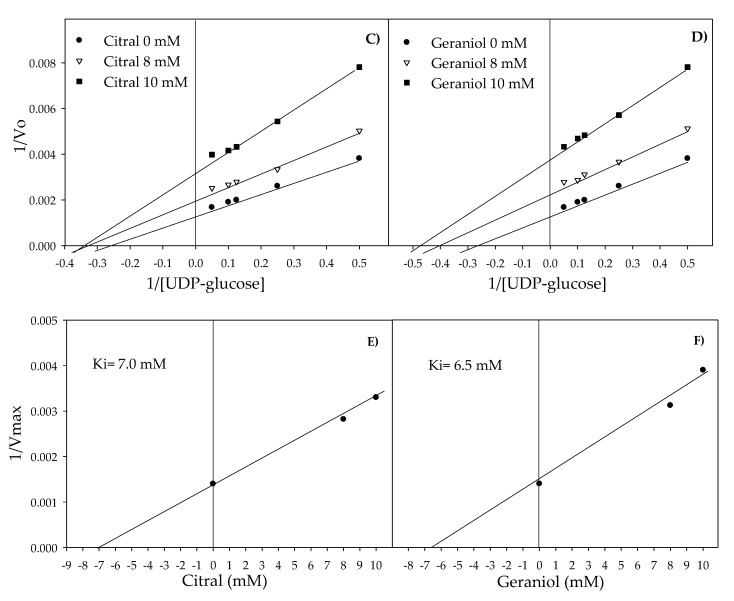
Reaction velocity of glucosyltransferase as a function of substrate concentration in the presence of citral (**A**) and geraniol (**B**). Lineweaver–Burk double reciprocal plot of the glucosyltransferase activity in the presence of citral (**C**) and geraniol (**D**). The double reciprocal plot of the glucosyltransferase activity as a function of citral (**E**) and geraniol (**F**) as a graphical method to calculate K_i_. Every point is a mean of three replicated experiments.

**Figure 6:**
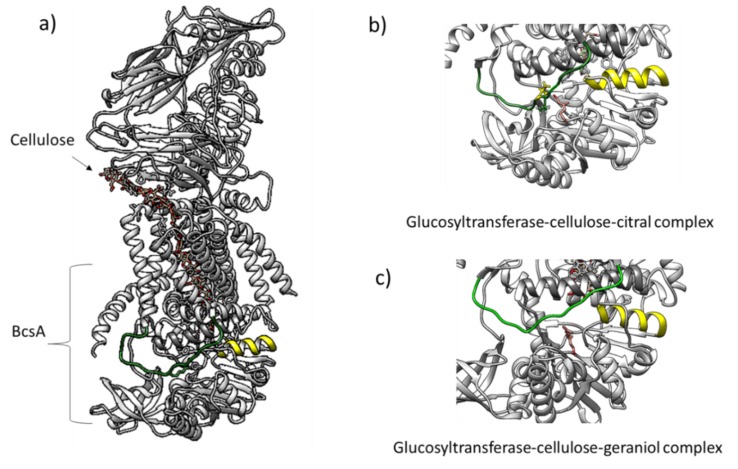
Glucosyltransferase (**a**) interactions with citral (**b**) and geraniol (**c**) blocking the gating loop (green) and the helix finger (yellow) movements during cellulose processing.

The activity of pure glucosyltransferase was affected by the presence of citral and geraniol showing IC_50_ values of 8.5 and 6.5 µM, respectively (Figure 4). The reaction pattern of the tested glucosyltransferase showed a Michaelis–Menten kinetic (Figure 5A,B). Table 1 shows the calculated kinetic constants, where both K_m_ and V_max_ decreased with increasing citral and geraniol concentrations (Figure 5C,D). On the other hand, low K_i_ values indicated that both inhibitors showed affinity towards the enzyme–substrate complex, this being higher in the case of geraniol. The steric arrangements that could explain the interference of terpenes were proposed by the computational docking analysis. Docking analysis showed that the most probable interactions among citral or geraniol and the enzyme occurred within the hydrophobic pocket located below the gating loop and next to the helix finger of the glucosyltransferase enzyme (Figure 6). The affinity energy obtained for the citral–enzyme-substrate complex was -5.8 kcal/mol with a root-mean-square deviation of atomic positions or RMSD 1.382 Å (Figure 6B), while for the geraniol–enzyme-substrate complex, it was -6.1 kcal/mol with RMSD 1.649 Å (Figure 6C).

## 3. Discussion

The contamination of food contact surfaces and the resistance of *E. coli* O157:H7 to disinfection processes are associated with its ability to form biofilms. The important role of glucosyltransferase producing glucans to strengthen the *E. coli* O157:H7 biofilms makes its inhibition an attractive target to reduce the biofilm formation. In this regard, *C. citratus* EO, citral, and geraniol have shown antimicrobial activities against many Gram-positive and Gram-negative bacteria, including *E. coli*. However, their effect on glucosyltransferase activity in relation with the biofilm formation has not been previously evaluated. 

*C. citratus* EO, citral, and geraniol inhibited the planktonic growth of *E. coli* O157:H7, and this effect could be attributed to their abilities to degrade membrane proteins and cell permeability. The higher antibacterial activity of citral and *C. citratus* EO compared with geraniol could be related to their hydrophobic characteristics, since they have partition coefficients (Log P) of 3 and 3.5, respectively [16,17], and these values could reflect a higher rate of interaction with the bacterial membrane. On the other hand, geraniol showed the lowest antibacterial activity against *E. coli* O157:H7, which may be explained considering its relatively lower lipophilic character (Log P = 2.9) [16] given by its hydroxyl group, which makes it more difficult to pass through non-polar environments such as the cell membrane [18] compared with citral and *C. citratus* EO. A similar situation was described for thymol (possess a hydroxyl group), which showed a lower efficacy against *E. coli* (MIC = 5 mg/mL) compared to p-cymene (absence of hydroxyl groups), which showed higher antibacterial activity (MIC = 2.5 mg/mL) [18]. 

Previously, Ortega-Ramirez et al. [10] reported the inhibitory effect of *C. citratus* EO against planktonic *E. coli* at 2.21 mg/mL. On the other hand, other EOs also showed efficacy to inhibit *E. coli* O157:H7; for example, Kim et al. [19] reported that concentrations of 0.001 to 0.01mg/mL of bay, clove, and pimento berry EO significantly inhibited the biofilm formation of *E. coli* O157:H7. Bazargani and Rohloff [20] reported an inhibition of *E. coli* O157:H7 adhesion of 72.3, 56.2, and 98.4% by coriander (1.6 mg/mL), anise (12.5 mg/mL), and peppermint EO (6.3 mg/mL), respectively. These results showed that *C. citratus* EO and its terpenes, citral and geraniol, showed efficacy as antibacterial agents inhibiting planktonic growth of *E. coli* O157:H7 even at low doses. It is important to mention that no previous reports of MBICs of these treatments were found in the revised literature; however, few mechanistic studies have been proposed. It is possible that the lower adhesion of the treated bacteria could be related to the interference in the adhesion process. Therefore, it has to be highlighted that the interest of this study was to evaluate the effect of *C. citratus* EO, citral, and geraniol on glucosyltransferase activity, glucan production, and biofilm development to propose a more complete mechanisms against cell communities that are the natural way of bacterial organization instead individual planktonic cells. For this reason, lower doses than MICs and MBICs were used to only affect the production of glucans without affecting cell viability.

Glucan production during *E. coli* O157:H7 biofilm formation was significantly reduced by citral and geraniol. Among the factors regulating the production of glucans in biofilms are the intercellular communication and the biosynthetic pathways [21]. Intercellular communication in *E. coli* occurs throughout the detection of acyl-homoserine lactones [22]; this process triggers the expression of virulence genes and the enzymatic production of glucans [23]. Thus, within the potential mechanisms of action of terpenes inhibiting glucans production are: (i) down-regulation of glucans synthase genes or a (ii) direct effect on the activity of such system [24]. Both approaches have been tested in other bacterial systems; however, most of the evidence has been directed to a possible effect on the enzymatic production of this polymer, as was done in the present study [24,25].

The ability of bacteria to adhere and form biofilms on different surfaces has substantial implications in the food industry due to safety, quality, and economic issues [26]. As mentioned above, the presence of glucans protects cells from the action of disinfectants and physical cleaning processes. In this sense, it is possible to use *C. citratus* EO, citral, and geraniol as alternative disinfectants to inhibit biofilm formation as well as to help enhance the effect of other cleaning methods. These data can be compared with previous studies that showed the efficacy of plant extracts and their active constituents to inhibit the production of water-insoluble glucans and biofilms of plaque-forming bacteria. Extracts of *Plectranthus barbatus*, *Plectranthus ecklonii*, and *Rheum undulatum* were effective in inhibiting the production of glucans in crude extracts of *Streptococcus sobrinus* and *S. mutans* [24]. 

For the same bacteria, Koo et al. [25] reported IC_50_ of 0.35 and 0.28 mg/mL for apigenin and farnesol, respectively. Also, epigallocatechin gallate, epigallocatechin, tannic acid, and catechol at 0.1 mg/mL inhibited the production of water-insoluble glucans of 73.1, 68.5, 68, and 67.6%, respectively [27]. In these studies, the reduction of glucans production was related with biofilm inhibition; however, most of them were done on dental plaque and tooth decay bacteria, not in a foodborne pathogen such as *E. coli* O157:H7. From the obtained results, it was observed that *C. citratus* EO, citral, and geraniol were effective in inhibiting the glucans production at non-lethal concentrations, maintaining their effect during the biofilm formation process.

*C. citratus* terpenes affected glucosyltransferase activity and, based on the obtained kinetic constants, this suggested an uncompetitive inhibition mechanism of glucosyltransferase by citral and geraniol, indicating that both terpenes bound reversibly to the enzyme–substrate complex, forming a ternary complex catalytically inactive. Citral and geraniol are molecules capable of accepting and donating hidrogens atoms and possess non-polar properties to establish hydrophobic interactions [16]. The interaction of terpenes within the hydrophobic pocket below the gating loop and the helix finger could affect the consequent UDP-glucose binding and glucan synthesis [6]. Cellulose synthase is activated by the presence of c-di-GMP, specifically by conformational changes caused by binding c-di-GMP, leading to an open state of the gating loop away from the active site cleft and near the water–lipid interface, where the loop is stabilized by the hydrophobic interactions with the BcsA’s amphipathic interface helices forming a transmembrane channel [6]. In this sense, the interruption of the helix finger movement by the presence of citral or geraniol affected the glucan polymerization by influencing the retraction and the insertion of the gating loop [6].

Although there is no evidence of the effect of plant extracts on the glucosyltransferase activity of *E. coli*, there are studies with the dental bacteria *Streptococcus* [24,25]. Plant extracts of *P. barbatus*, *P. ecklonii*, and *R. undulatum* inhibited the activity of glucosyltransferase in crude extracts of *S. sobrinus* (IC_50_ = 1.0, 1.2 and 0.142 mg/mL, respectively) and *S. mutans* (IC_50_ = 3.1, 1.6 and 0.079 mg/mL, respectively) [24]. Within the same study, rosmarinic acid, one of the main components of these plants, showed IC_50_ of 2.1 and 3.9 mg/mL for *S. sobrinus* and *S. mutans* enzyme extracts, respectively. However, these studies did not propose any inhibition mechanism. On the other hand, oleic and linoleic acids showed to be uncompetitive inhibitors of glucosyltransferase; these fatty acids interacted with the substrate–enzyme complex, decreasing the velocity reaction in a similar way to that observed with *C. citratus* EO terpenes [28].

*C. citratus* EO and its components also inhibited the activity of other enzymes; for example, *C. citratus* EO inhibited MARK4, a kinase enzyme involved in apoptosis, inflammation, and many other regulatory pathways [14]. In another study, seven monoterpenes of *C. citratus* EO were evaluated on pentoxyresorufin activity, obtaining IC_50_ of 0.087 mM for (-)-α-pinene, 0.089 mM for (+)-α-pinene, 0.76 mM for α-terpinene, and 1.19 mM for citral [29]. For this reason, it is important to consider the effect of the rest of the EO components against glucosyltransferase activity, glucan production, and biofilm inhibition of *E. coli* O157:H7. As shown in previous studies, there is evidence that *C. citratus* EO and its compounds were capable of inhibiting different enzymes, but there was no evidence of the effect of this EO against *E. coli* O157:H7 biofilm-glucans-glucosyltransferase, which is the contribution of this study.

## 4. Material and Methods

### 4.1. Susceptibility of Planktonic and Biofilm E. coli O157:H7 Cells to C. citratus EO, Citral, and Geraniol

The antibacterial efficacies of *C. citratus* EO (W523100), citral (W230316), and geraniol (W250716) (Sigma-Aldrich, St. Louis, MO, USA) were evaluated against the growth of planktonic and biofilm *E. coli* O157:H7 (ATCC 43890). MIC experiments were performed by the broth microdilution method reported by the Clinical and Laboratory Standards Institute or CLSI [30] with some modifications. Briefly, 5 μL of an overnight inoculum of *E. coli* O157:H7 (1 × 10^6^ CFU/mL) diluted in sterile saline solution were added to a sterile 96-well microtitre plate (Costar 96, Sigma-Aldrich, St. Louis, MO, USA), followed by 295 µL of EO, citral, and geraniol diluted in Luria Bertani o MH (LB) broth at concentrations from 1 to 20 mg/mL, obtaining 2-fold dilutions, respectively. The microplate was incubated at 37 °C for 24 h, and the MICs were determined as the lowest concentrations of each agent that completely inhibited the visible growth of planktonic cells. 

For inhibiting biofilm bacteria, MBICs were determined as the lowest dose of each compound inhibiting the bacterial adhesion on stainless steel coupons (1 × 1 × 0.1 cm, grade 304) during 24 h of incubation at 37 °C [31]. Different concentrations of natural compounds (0–20 mg/mL) were added into test tubes with 10 mL of MH broth containing stainless steel coupons. Then, the tubes were inoculated with *E. coli* O157:H7 (1 × 10^6^ CFU/mL, diluted in sterile saline solution) and incubated at 37 °C for 24 h under static conditions; then, the coupons were removed from the culture medium and washed with sterile distilled water to remove weakly adhered cells. Afterward, the coupons were placed in 5 mL of sterile peptone water and subjected to an ultrasonic bath (40 kHz) for 5 min to release the strongly adhered cells and were counted by plating on MH agar after 24 h of incubation at 37 °C (log CFU/cm^2^). Both inhibitory concentrations were obtained by triplicate from three independent experiments, and the obtained results were expressed as mg/mL [31].

### 4.2. Effect of C. citratus EO, Citral, and Geraniol on the Glucans Content in E. coli O157:H7 Biofilms

Lower doses than MICs and MBICs were used to only affect the production of glucans without affecting cell viability. The conditions used for biofilm formation were as described above; applying *C. citratus* EO (0.5 mg/mL), citral (0.5 mg/mL), and geraniol (0.25 mg/mL), viable cells were counted at different times (0, 2, 4, 8, 10, 12 h) at 37 °C. Biofilm cells adhered to stainless steel coupons as well as planktonic cells in the culture medium were determined as described above, expressing results as log CFU/cm^2^ and log CFU/mL, respectively. Also, biofilms were stained with 0.1% crystal violet solution for 10 min and fixed with Lugol to observe morphological changes during the exposure to the treatments using an inverted microscope (Zeiss Axio Vert A1 Inverted, Carl Zeiss, NY, USA), viewing with phase contrast at 600× [32].

The glucans production by treated bacteria was expressed as glucose equivalents (GE) per area of stainless steel (cm^2^) [32]. Coupons were removed from the culture medium after incubation and then washed with water to remove weakly adhered cells. Subsequently, they were placed into tubes containing 5 mL of water and 30 μL of formaldehyde (33%) (Sigma Aldrich, St. Louis, MO, USA) and left at 4 °C for 1 h. Subsequently, 2 mL of NaOH (1 M) (Sigma Aldrich, St. Louis, MO, USA) were added to the tubes, sonicated for 5 min, and stored for 3 h at 4 °C. The final volume (7 mL) was filtered (millipore 0.22 μm) and dialyzed with Milli-Q water using a dialysis membrane (3500 Da) (Sigma Aldrich, St. Louis, MO, USA) at 4 °C for 24 h, and the > 3500 Da fraction was lyophilized. The lyophilized sample was diluted in 300 μL of Milli-Q water for the subsequent quantification of glucans adhered to the stainless steel coupons. The glucans were determined with the phenol/sulfuric acid method [33] using glucose as standard and expressing results as mg of glucose equivalents per area, GE/cm^2^.

### 4.3. Inhibition of Glucosyltransferase Activity by Citral and Geraniol

Glucosyltransferase (SRP0416, Sigma Aldrich, St. Louis, MO, USA) activity was measured in the presence of citral and geraniol at 0, 8, and 10 μM; lemongrass EO was excluded from this assay considering the variety of chemical structures in its content, making it difficult to establish a molar relation. This was measured in 300 μL of buffer solution (40 mM Tris-HCl, pH 8, 15 mM MgCl_2_, 1 mM CaCl_2_, and 5 mM UDP-glucose) containing each concentration of terpenes; this mixture was pre-incubated at 30 °C for 10 min, and the reaction was initiated by adding the glucosyltransferase (EC 2.4.1.). The enzyme activity was measured using the fluorometric assay [34] that monitored the release of UDP-fluorescein (λ_ex_ 490 nm; λ_em_ 514 nm) as a product of the UDP-glucose hydrolysis (the absence of terpenes in the reaction was taken as 100% activity). 

The initial reaction velocities (V_o_) were obtained using 2 mM of glucosyltransferase, substrate at 2, 4, 8, 10, and 20 μM, and the individual terpenes at 8 and 10 μM, respectively. The experimental data were fitted to a non-linear model, applying the equation of Michaelis–Menten for K_m_ and V_max_ calculation, and then these values were fitted to the Lineweaver–Burk equation. The type of inhibition was determined analyzing the Lineweaver–Burk graph, and the K_i_ values of the individual terpenes were taken from the x-intercepts of 1/V_max_ versus the terpene concentration [35]; this assay was performed three times to assure reproducibility. 

### 4.4. Molecular Docking of Glucosyltransferase with Citral and Geraniol

Molecular docking was used to identify possible interactions between the individual terpenes (citral and geraniol, respectively) with the glucosyltransferase crystallographic model (PDB 5EIY) [6]; the used citral and geraniol models were PubChem 638011 and PubChem 637566. This analysis was done using the AutoDoc Vina application in the UCSF Chimera version 1.13 software (Resource for Biocomputing, Visualization, and Informatics, University of California, San Francisco, CA, USA) to obtain affinity energies (kcal/mol) with the lowest root-mean-square deviation (RMSD, Å) between glucosyltransferase and each terpene. Ten binding modes with a 3 level of exhaustiveness search and a 3 kcal/mol level of maximum energy difference were set as basic parameters during the analysis.

### 4.5. Statistical Analysis

A completely randomized experimental design was done for all assays. The effect of *C. citratus* EO, citral, and geraniol, as well as the exposure time (0, 2, 4, 8, 10, and 12 h) were evaluated on the count of viable planktonic and biofilm cells and the glucans production. In addition, a Pearson correlation was done between the secreted glucans and the biofilm formation. All experiments were done by triplicate, expressing the results as means ± standard deviation. An analysis of variance (ANOVA) was done for all the assays to estimate significant differences among treatments, and the means were compared by the Tukey–Kramer test. All experiments were performed at *p* ≤ 0.05 using the statistical software NCSS 2007 (NCSS, LLC, Utah, USA).

## 5. Conclusions

*C. citratus* EO, citral, and geraniol were capable of inhibiting *E. coli* O157:H7 biofilm formation, decreasing cell adhesion and glucans production on stainless steel surfaces. This inhibitory effect could be related to an uncompetitive inhibition of glucosyltransferase activity caused by the presence of citral and geraniol. These results suggest a possible inhibition mechanism of terpenes on biofilm formation of *E. coli* O157:H7.

## Figures and Tables

**Table 1:** Kinetic parameters of glucosyltransferase exposed to citral and geraniol.

Agent	Concentration (µM)	K_m_ * (µM)	V_max_ * (µmol UDP**-glucose min/mL)	Ki (µM) *
Citral	0	3.42	714.28	
	8	2.66	476.19	7
	10	2.66	303.03	
Geraniol	0	3.42	714.28	
	8	2.20	416.66	6.5
	10	2	256.41	

* Values are means of three replicated experiments. ** UDP: Uridine diphosphate.
